# In silico assessment of nanoparticle toxicity powered by the Enalos Cloud Platform: Integrating automated machine learning and synthetic data for enhanced nanosafety evaluation

**DOI:** 10.1016/j.csbj.2024.03.020

**Published:** 2024-03-30

**Authors:** Dimitra-Danai Varsou, Panagiotis D. Kolokathis, Maria Antoniou, Nikolaos K. Sidiropoulos, Andreas Tsoumanis, Anastasios G. Papadiamantis, Georgia Melagraki, Iseult Lynch, Antreas Afantitis

**Affiliations:** a NovaMechanics MIKE, Piraeus 18545, Greece; b Entelos Institute, Larnaca 6059, Cyprus; c NovaMechanics Ltd, Nicosia 1070, Cyprus; d School of Geography, Earth and Environmental Sciences, University of Birmingham, B15 2TT Birmingham, UK; e Division of Physical Sciences and Applications, Hellenic Military Academy, Vari 16672, Greece

**Keywords:** Nanoinformatics, Synthetic data, Automated machine learning, Safety and sustainability by design

## Abstract

The rapid advance of nanotechnology has led to the development and widespread application of nanomaterials, raising concerns regarding their potential adverse effects on human health and the environment. Traditional (experimental) methods for assessing the nanoparticles (NPs) safety are time-consuming, expensive, and resource-intensive, and raise ethical concerns due to their reliance on animals. To address these challenges, we propose an *in silico* workflow that serves as an alternative or complementary approach to conventional hazard and risk assessment strategies, which incorporates state-of-the-art computational methodologies. In this study we present an automated machine learning (autoML) scheme that employs dose-response toxicity data for silver (Ag), titanium dioxide (TiO_2_), and copper oxide (CuO) NPs. This model is further enriched with atomistic descriptors to capture the NPs’ underlying structural properties. To overcome the issue of limited data availability, synthetic data generation techniques are used. These techniques help in broadening the dataset, thus improving the representation of different NP classes. A key aspect of this approach is a novel three-step applicability domain method (which includes the development of a local similarity approach) that enhances user confidence in the results by evaluating the prediction’s reliability. We anticipate that this approach will significantly expedite the nanosafety assessment process enabling regulation to keep pace with innovation, and will provide valuable insights for the design and development of safe and sustainable NPs. The ML model developed in this study is made available to the scientific community as an easy-to-use web-service through the Enalos Cloud Platform (www.enaloscloud.novamechanics.com/sabydoma/safenanoscope/), facilitating broader access and collaborative advancements in nanosafety.

## Graphical Abstract

**Figure:**
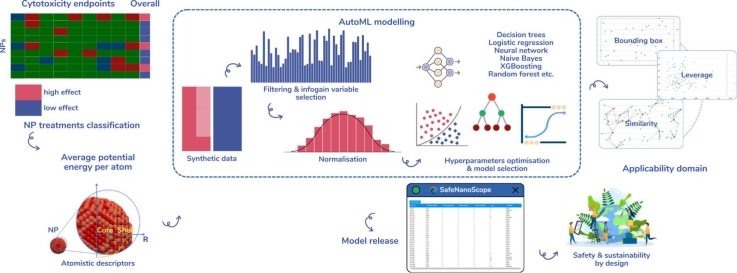

## 1 Introduction

The intriguing properties of nanoparticles (NPs), in comparison to the same material in macroscale, have fuelled the growing use of NPs in commercial products [1]. At the time of writing, NPs are incorporated in more than 5300 commercial products worldwide [2], and the global NP market, valued at $16.3 billion in 2021, is projected to reach $62.8 billion by 2031 [3]. During their life-cycle, nano-enabled products may release NPs to the environment and through different exposure mechanisms (e.g., inhalation, injection, dermal exposure, via the food chain) they may reach and cause adverse effects to different organisms [4], [5]. Upon release into the environment, NPs are subject to transformations (e.g., biocorona formation, agglomeration, dissolution, morphological and surface charge alterations etc.) that may alter their physicochemical parameters and thus, their overall fate and behaviour [6], [7], [8].

In line with the European Green Deal’s principles, nanomaterials entering the EU market, along with other materials and chemicals, are expected to adhere to the safety and sustainability by design (SSbD) framework. This comprehensive approach aims to minimise the negative impacts of these products and substances on human health and the environment through early consideration and design-out of potentially harmful aspects. Within the SSbD framework, development of novel approach methodologies (NAMs) is encouraged to effectively generate data and assess NPs in the nanosafety field. These NAMs aim to screen and filter out potentially unfavourable candidate NPs as early as possible in the R&D process, paving the way for the design and redesign of novel and safer NPs [9]. Moreover, NAMs comply with the 3 R (Replacement, Reduction, and Refinement) principles [10] which aim to reform research towards more ethical practices, including non-animal methods such as in vitro and *in silico* approaches. The combination of different NAMs, both experimental and computational, under an integrated approaches to testing and assessment (IATA) framework will additionally facilitate the complete risk assessment of NPs and accelerate the regulatory decision-making processes [9], [11], [12].

The nanoinformatics field encompasses different computational methodologies and data science approaches to assess the risks and hazards associated with NPs in both short and long-term exposure scenarios. Examples include virtual screening strategies for prioritising NPs with desired properties for further experimental evaluation, and the development of quantitative structure-activity relationship models tailored to the unique properties of materials at the nanoscale (nanoQSAR). Physiologically based pharmacokinetic (PBPK) modelling and molecular dynamics simulations further deepen our understanding of NP behaviour and interactions. Additionally, grouping and read-across strategies are developed to predict NPs properties even when limited data are available [11], [13], [14].

*In silico* nanoQSAR-type and read-across models serve as alternative to the conventional experimental approaches for the assessment of the adverse effects of NPs [11], [14]. Various computational methodologies [15], [16], [17] and predictive models [18], [19], [20] have been proposed over the past years for the prediction of different NP properties and toxicity endpoints. The integration of automation and optimisation methodologies [21], [22], [23] and of ensemble learning approaches [24], [25] into the computational assessment of NP properties, as well as the incorporation of recent advances of artificial intelligence (AI) and machine learning (ML), drive the development of models with improved features for increasingly accurate predictions. For example, deep learning methodologies have been proposed for the classification of the NP effects, bio-interactions or other properties based on sets of experimentally derived images of organisms exposed to NPs or from nanostructure images [26], [27].

Automation of the ML model development process is a significant challenge but will improve modelling accuracy and efficiency. Automated ML (autoML) schemes have already been applied in material design studies [28] and lately to nanotoxicity datasets, to achieve optimised results and high accuracy in the prediction of the query endpoint (in comparison to the standard nanoQSAR development workflows)[29], [30]. Automated nanoinformatics methodologies have also been proposed for the automatic selection of the best-performing approach between NPs grouping or linear regression methodologies[31], and for the selection of the best performing model from a pool of available regression and/or classification methodologies [32], [33]. Such automated methodologies save time and resources during the *in silico* investigation of NPs, allowing researchers to focus on the interpretation of the respective results.

One of the most demanding challenges in the development of ML-based nanoinformatics is the scarcity of NP data and metadata [34] and/or dataset imbalance, i.e., where too many of the NPs in the set are non-toxic for example. These issues hinder firstly the development of reliable models, and secondly, their regulatory acceptance through evaluation with external data [13]. Despite various modelling approaches and algorithms being proposed, they are validated on a limited number of curated datasets, thus their effectiveness in everyday applications has yet to be extensively tested. To mitigate this, data enrichment strategies are employed to incorporate data that could increase the value of existing sets by adding information from density functional theory (DFT) calculations [19], [35], by determining periodic table [36], [37] and image analysis [38], [39] descriptors, and by collating information from different data sources [29], [37]. In addition, data imbalances are addressed using reliable oversampling techniques that generate synthetic data samples, ensuring equal class representation in the datasets [40], [41].

From the pool of NPs used in commercial products, our work is focused on silver (Ag), titanium dioxide (TiO_2_), and copper oxide (CuO) NPs, selected for their unique properties and wide range of applications. Ag NPs have excellent antibacterial and antifungal properties meaning that they can be used to kill bacteria and fungi on surfaces, making them useful for a variety of applications, including in medical devices, food packaging, and textiles [42]. TiO_2_ NPs have applications in coatings, inks and paints, suncreams, toothpastes, food colourants, and wastewater treatment [43]. They have a relatively wide band gap (e.g., band gap energy 3.2 eV for anatase) which means they absorb ultraviolet (UV) light [44]. Absorption of visible light can be maximised by functionalisation of TiO_2_ NPs with organic and inorganic materials, thereby enhancing the photocatalytic properties under sunlight [45]. CuO NPs were selected due to their semiconductor nature, which makes them important for solar cells, batteries, and sensor applications [46]. CuO NPs also have high catalytic activity, and antimicrobial activity and are widely used in environmental applications [47].

The presented work integrates in vitro experimental data with atomistic descriptors derived from computational methodologies, to build a ML model for the prediction of the adverse effect class of Ag, TiO_2_, and CuO NPs. In this way, different data manipulation strategies were combined first to adjust the underrepresentation of one of the endpoint classes by generating synthetic data, and second to perform an optimised modelling including data cleansing and variable selection through an autoML scheme. The selected descriptors are discussed to highlight the influence of each one on the toxicity endpoints assessed which include cell viability, mitochondrial membrane potential and nuclear size (see Table 1). Care is also taken to support the generated predictions with a well-defined applicability domain (AD). For this purpose, a novel AD strategy is used that considers two well-known AD methodologies (the bounding-box and the leverage approach), combined with a local similarity approach proposed here for the first time. According to this strategy, the reliability of each prediction can be classified as “good”, “moderate”, or “poor” based on a weighted scheme that incorporates the results of the three above-mentioned strategies. Finally, to further enhance end-user confidence in our model, both the data and the model, and their associated metadata, are made publicly accessible. The data is available through the nanoPharos database, and the model can be accessed via the Enalos Cloud platform.

**Table 1:** List of measured endpoints utilising the HTS-HCI screening [49].

Measured endpoints of the HTS-HCI screening
Valid Object Count
Cell Viability
Reduced Mitochondrial Potential
Cell Membrane Damage
Nuclear size
Nuclear Intensity
Attached cells without reduced mitochondrial potential and no cell membrane damage
Attached cells with reduced mitochondrial potential but without cell membrane damage
Attached cells with normal mitochondrial potential but with cell membrane damage

## 2 Data

The nanoinformatics models developed in this study used data on the in vitro toxicity of Ag, TiO_2_, and CuO NPs, generated via the NanoMILE project [48], as described in detail by Joossens *et al*. [49]. Specifically, the human hepatoma HepaRG cell line was treated with 89 different NPs across 10 concentrations. The study employed High Throughput Screening (HTS) combined with High Content Imaging (HCI) to classify NP hazards and identify candidates for further toxicological assessment. In the present study, endpoint and dose-response data on 11 Ag, TiO_2_, and CuO NPs was used encompassing a total of 110 treatments (combinations of NP samples and concentrations). The NPs’ size, shape, and phase (for TiO_2_ NPs) information were derived from the NanoMILE deliverables, reports, and relevant publications [50], [51] (see Table 2).

**Table 2:** Information on the assessed NPs: Size, shape and phase data were retrieved directly from the NanoMILE deliverables. The “Size in ASCOT” and the FF of the last column were used to calculate the computational descriptors [49], [50], [51].

NanoMILE ID	NP	Metal core	Coating	Size [nm]	Size in ASCOT [nm]	Shape group	FF using OPENKIM ID[57]
NP00214	Ag (JRC NM-300 K) pristine	Ag	Coated	21.097	21.097	Spherical	EAM_Dynamo_ AcklandTichyVitek_ 1987v2_Ag__MO_ 055919219575_000[58]
NP00432	AgPURE_15nm	Ag	Coated	19	19	Spherical	EAM_Dynamo_ AcklandTichyVitek_ 1987v2_Ag__MO_ 055919219575_000
NP00255	TiO2 uncoated (PROM)	TiO_2_ Anatase	Uncoated	Longest: 8.0 Shortest: 11.9	9.95	Spherical	Sim_LAMMPS_ MEAM_ZhangTrinkle_ 2016_TiO__SM_ 513612626462_000[59]
NP00256	TiO2-PVP pristine (PROM)	TiO_2_ Anatase	Coated	Longest: 11.86 Shortest: 8.99	11.65	Various shapes: Mainly square	Sim_LAMMPS_ MEAM_ZhangTrinkle_ 2016_TiO__SM_ 513612626462_001
NP00257	TiO2-F127 (PROM)	TiO_2_ Anatase	Coated	Longest: 13.4 Shortest: 10.4	13.32	Faceted/square	Sim_LAMMPS_ MEAM_ZhangTrinkle_ 2016_TiO__SM_ 513612626462_002
NP00258	TiO2 AA4040 (PROM)	TiO_2_ Anatase	Coated	Longest: 13.9 Shortest: 9.9	13.24	Faceted/square	Sim_LAMMPS_ MEAM_ZhangTrinkle_ 2016_TiO__SM_ 513612626462_003
NP00259	TiO2 (JRC NM-103)	TiO_2_ Rutile	Uncoated	Longest: 40.7 Shortest: 25.3	X: 40.7 Y: 25.3 Z: 25.3	Nanorods	Sim_LAMMPS_ MEAM_ZhangTrinkle_ 2016_TiO__SM_ 513612626462_000
NP00260	TiO2 (JRC NM-104)	TiO_2_ Rutile	Uncoated	Longest: 42.5 Shortest: 23.2	X: 42.5 Y: 23.2 Z: 23.2	Nanorods	Sim_LAMMPS_ MEAM_ZhangTrinkle_ 2016_TiO__SM_ 513612626462_000
NP00441	TiO2D540 10 nm (PROM)	TiO_2_ Anatase	Coated	Longest: 10.8 Shortest: 8.2	9.5	Spherical	Sim_LAMMPS_ MEAM_ZhangTrinkle_ 2016_TiO__SM_ 513612626462_000
NP00458	CuO_CuO360 (UoB)	CuO	Coated	12.144	12.144	Spherical	Sim_LAMMPS_ IFF_PCFF_ HeinzMishraLinEmami_ 2015Ver1v5_ FccmetalsMinerals SolventsPolymers__ SM_039297821658_000[60], [61], [62]
NP00456	CuO_CuO10 (UoB)	CuO	Coated	5.95	5.95	Spherical	Sim_LAMMPS_ IFF_PCFF_ HeinzMishraLinEmami_ 2015Ver1v5_ FccmetalsMinerals SolventsPolymers__ SM_039297821658_000

The cytotoxicity experiments assessed the cell viability and mitochondrial health by measuring 9 toxicity features/endpoints (Table 1). The results of the HTS-HCI screening were normalised following the signal-to-noise ratio approach. This normalisation process enabled comparison of data from different features and experiments on a single plot, and it provided an indication of whether the effect induced by a test item (a specific NP and concentration) was statistically significant relative to the untreated (negative) control. A threshold of − 3 for downward response and + 3 for upward response was used, which corresponded to a 99% certainty of cell behaviour deviation from the untreated control (cells treated only with medium). The normalised values were presented in a colour-coded heatmap, which reflected the degree of difference in behaviour from the control (red and blue for decreased or increased response, respectively) or indicated that the response was similar to that of the negative control (green colour)[49].

For the *in silico* toxicity assessment of NPs on the HepaRG cell line, the results of the 9 toxicity features were summarised into a single endpoint (“overall”) class. NP treatments were labelled as “low effect” if they exhibited a response similar to the negative controls (green labels) in at least 5 of the measured features (73 NP treatments). Otherwise, NP treatments were classified as “high effect” meaning that their response differed significantly from the negative controls (37 treatments).

### 2.1 Data enrichment

As NP experimental data for the development of ML models are still limited and fragmented across different sources and formats[34], data enrichment strategies have emerged to supplement the available information and increase its value[52] (e.g., inclusion of periodic table descriptors[36], image descriptors[38], theoretical descriptors[19] etc.). In the present work, the dose-response dataset was enriched with atomistic descriptors using the ASCOT software (https://www.enaloscloud.novamechanics.com/sabydoma/ascot/)[53], [54] developed as part of the Sabydoma project[55]. To conduct the simulations and obtain the computational descriptors, information on the size, shape, and phase of the NPs was required as presented in Table 2, as well as information on the used force field (FF). The FF is a mathematical function of the position of the system’s atoms which is used to estimate the forces acting between them. It is important to note that the ASCOT software calculates descriptors solely for the NPs core in vacuum, implying that the generated atomistic descriptors refer to uncoated NPs. Additionally, the software can compute descriptors only for spherical and ellipsoid NPs (see ASCOT extended version http://enaloscloud.novamechanics.com/riskgone/nanoconstruct/). For non-spherical NPs, the following assumptions were made:

• Square/faceted NPs were treated as spheres with a diameter equal to the particles’ equivalent sphere area diameter[56].
• Rod-shaped NPs were modelled as ellipsoid NPs. In this representation, the X-axis dimension equals the NPs longest diameter, and the Y and Z axes dimensions equal the NPs shortest diameter. Calculations for ellipsoid NPs involved three different rotation angles on the Z-axis (30°, 60° and 90°). The ellipsoid configuration yielding the minimum average potential energy of all atoms was selected as the most stable structure.

The calculated descriptors were collated into a spreadsheet including only the descriptors common to both spherical and ellipsoid NPs. A detailed list of these descriptors is available in the supporting information file of this publication. To enhance data re-usability the complete dataset is accessible through the nanoPharos database (https://db.nanopharos.eu/Queries/Datasets.zul?datasetID=16).

## 3 Methods

### 3.1 General autoML workflow

**Fig. 1:**
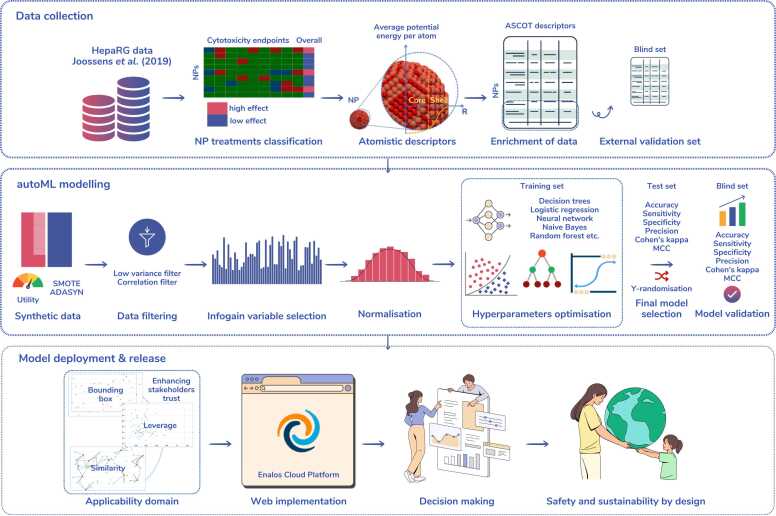
Schematic workflow of the data analysis, modelling of the toxicity endpoints, and release of the final model.

The collected data (NP characteristics, treatments and classification, enriched with atomistic descriptors) is investigated *in silico*, by developing ML models that could predict the adverse effects of NPs from their computational descriptors. This work demonstrates the potential of fully *in silico* NAMs approaches, whereby NPs toxicity could be screened based on their proposed compositions prior to their actual synthesis, and therefore contributes to the SSbD of novel NPs by screening out toxic NPs at the earliest possible stage. It is noted that the modelling is performed considering the OECD principles for the validation of QSAR models (a defined endpoint, an unambiguous algorithm, a defined domain of applicability, appropriate measures of goodness-of-fit, robustness and predictivity, and a mechanistic interpretation, if possible) [63]. To begin with, a subset of the data is randomly selected using a stratified sampling technique and excluded from the model development process, to later serve as a blind set for validation purposes. The remaining data is fed to the autoML workflow and is split again into training and test sets; the training set is used for model development whereas the test set is used to select the best performing model inside an autoML scheme. The training data is oversampled to balance the relative frequency of the two endpoint classes (“low effect” and “high effect”). Later, data is filtered and fed into the core of the autoML modelling where the best performing model among seven tested and optimised algorithms (as listed in Table 3) is selected as the final model. The best performing model is applied on the blind set to assess the model’s performance in real conditions. The applicability domain of the model is assessed using three different techniques and the model is released as a web-service through the Enalos Cloud Platform. The overall analysis is schematically presented in Fig. 1 and the individual steps are explained in detail in the next paragraphs. This information is also provided in a more straightforward manner through a QSAR model reporting format (QMRF) report in the supplementary information files of this publication.

**Table 3:** List of assessed ML methodologies in the autoML methodology.

ML methodologies	Optimised hyperparameters
Gradient boosted trees	Number of trees
Naïve Bayes	Default probability
Logistic regression	Step size
Decision tree	Minimum number of records per node
Random forest	Tree depth, number of trees, minimum child node size
Neural network	Number of hidden layers, number of hidden neurons per layer
XGBoost trees	Eta, max depth

### 3.2 Synthetic data

Class imbalance may prevent the generalisation of the models, creating bias over the majority class and leading to poor classification performance. To address the issue of imbalance between the two classes (66% “low effect” vs. 34% “high effect” treatments in the initial dataset also reflected in the subsets), we apply two oversampling techniques in the underrepresented class, namely the synthetic minority over-sampling technique (SMOTE) [64] and the adaptive synthetic sampling approach for imbalanced learning (ADASYN)[65]. In the SMOTE, which has also been used in other cases of nanoinformatics ML models [24], [25], [40], [41], [66], [67], the training data are augmented with “high effect” treatments by selecting a treatment and one of its *k-*nearest neighbours (*k*NN) and drawing a new synthetic treatment along the line between the two original treatments. In the SMOTE implementation used in this work, the generated table contained the same number of treatments for each of the classes [68]. The ADASYN approach, on the other hand, generates synthetic treatments of the underrepresented class for original treatments that are more difficult to learn because they are closer to the majority class treatments in the hyperspace. Again, the *k*NN method is used for the synthetic data generation, through the respective R function [69]. In this work the oversampling of the underrepresented class is performed only in the training set, so that the subsequent validation is not affected by oversampling. The training data are transformed using z-score normalisation, so that the *k*NN method, that employs Euclidean distances, is not affected by the differences in descriptors ranges. After synthetic data generation the training data are denormalised. Furthermore, to ensure consistent results, interdependent descriptors (such as descriptors expressing ratios and differences of other descriptors) do not participate in the synthetic treatments generation, but they are recalculated after the application of SMOTE and ADASYN and the denormalisation of the data. Columns with missing values are also excluded prior to the synthetic data generation.

While synthetic data offer a fast and cost-effective approach to augmenting nano-datasets, it is crucial to ensure that the generated data is suitable for modelling, to ensure that the resulting predictions are meaningful and reliable. To ensure the appropriateness and compatibility of the synthetic data produced both with SMOTE and ADASYN with the original data, we assess the utility of the synthetic data using the propensity score mean square error (pMSE). This metric quantifies the extent to which the synthetic treatments can be distinguished from the original ones. For the oversampled class (“high effect” class in our case) a binary indicator is assigned to original (0) and synthetic (1) treatments. Next a logistic regression model is built based on the leave-one-out (LOO) cross-validation method (including variable filtering and normalisation) to discriminate between real and synthetic data points. The predicted probability values ($\hat{p_{\iota }}$) for each treatment are recorded and the pMSE is then calculated as per Eq. 1[70], [71].

(1) $$\mathrm{pMSE}=\frac{1}{N}\sum_{i=1}^{N}{\left(\hat{p_{i}}-c\right)}^{2}$$

where, N is the number of NP treatments after oversampling (combination of original and synthesised data), $\hat{p_{\iota }}$ are the predicted probabilities for synthetic data, and c denotes the ratio of synthetic data in the N.

Our goal for the oversampled treatments is to ensure that they will be as similar to the original treatments as possible. Thus, in this classification model, the desired output is poor classification [70] and consequently values of the pMSE closer to zero indicate lower distinguishability (greater similarity) between the two sets [71]. The pMSE values are calculated considering all treatments after oversampling performed with SMOTE and ADASYN and the method that produces the most similar data to the original (lower pMSE value) is selected for the rest of the analysis.

To strengthen user confidence in the selected oversampling method between SMOTE and ADASYN, the Kullback-Leibler (KL) divergence [72] and Wasserstein distance [73] metrics are calculated based on the descriptors relative frequency distributions. These metrics quantify the similarity between two probability distributions, with lower values indicating greater resemblance. In this context, KL divergence and Wasserstein distance values closer to zero between the original and oversampled training sets suggest higher similarity and potentially more reliable model predictions.

### 3.3 Data pre-processing

After the generation of the synthetic treatments (i.e., the oversampling of the “high effect” class using SMOTE and ADASYN), data is pre-processed to exclude non-informative descriptors, to avoid over-fitting phenomena, and to improve the algorithms’ performance. For this reason, a low-variance filter is applied on the training data using a minimum variance threshold of 0.2 for the input descriptors. Next, to reduce the redundancy in input information that can confuse the model and lead to poor generalizability, for each pair of descriptors the Spearman’s rank correlation coefficient is calculated to measure the intensity of a monotonic relationship between them, and later using a threshold value for the correlation coefficient (0.95 in this case) the redundant descriptor columns are filtered out. In this way, interdependent descriptors are removed from the dataset and a possible source of poor model performance on unseen data is minimised, as well. Finally, the information gain [74] of all remaining descriptors is calculated and descriptors with zero information gain score are excluded from the modelling, as they are not considered critical for establishing a predictive relationship. Thus, the final model is made more interpretable. After the above filtering steps, the training set is reviewed for possible duplicate treatments that occurred from the filtering of descriptors. Finally, the selected training descriptors are normalised using the z-score (Gaussian) method [75], to ensure their equal contribution to the analysis. The same normalisation functions are later applied to the test and blind sets.

### 3.4 Model development

After data preprocessing, it is fed into the core of the autoML scheme where models are trained to predict the NPs adverse effect class against HepaRG cells, using seven different methodologies (Table 3). The methodologies hyperparameters are tuned and the generated models are validated to assess the reliability of the predictions on the test data and to select the best performing one. More specifically, the training set is used to train the seven models and through a five-fold cross validation scheme applied to each of the models, a set of hyperparameters is optimised (Table 3). The optimisation is performed in an iterative manner: a combination of the values of the model hyperparameters is selected first through random search and the model is trained using the five-fold cross-validation. For each of the five trained variants of the specific model (from the five-fold cross validation) the Cohen’s kappa metric (Eq. 2) is recorded and averaged into a single value. This process is repeated for a specific number of iterations (10 in this case) or until the average Cohen’s kappa is not improved after 5 iterations. Each of the seven models is finally tuned with the combination of hyperparameters leading to the highest average Cohen’s kappa value. Later, the seven tuned models are applied to the test set treatments and their performance is encoded in different statistical metrics (e.g., accuracy, precision, recall, specificity, Cohen’s kappa, and Matthews Correlation Coefficient (MCC))[76]. The best-performing model is selected based on the values of these metrics.

### 3.5 Model validation

The developed model (selected after optimisation via the autoML scheme as described above) is validated externally using the blind set treatments that do not participate in the model development. The blind set treatments are randomly selected from the original set of NP treatments prior to any modelling steps using a stratified approach, to retain the distribution of classes in the blind set. The performance of the model is evaluated based on the number of correct predictions and the number of misclassifications, by calculating the sensitivity, the specificity, the accuracy, the precision, the MCC and the Cohen’s kappa metrics using the formulas presented in Table 4.

**Table 4:** List of metrics used for the validation of the classification models.

Metric	Formula
Cohen’s kappa	$\kappa =\frac{2(\mathrm{TP}\times \mathrm{TN}-\mathrm{FP}\times \mathrm{FN})}{\left(\mathrm{TP}+\mathrm{FP}\right)\left(\mathrm{FP}+\mathrm{TN}\right)+(\mathrm{TP}+\mathrm{FN})(\mathrm{TN}+\mathrm{FN})}\;$(2)
Sensitivity or Recall (True Positive Rate)	$\mathrm{TPR}=\frac{\mathrm{TP}}{\mathrm{TP}+\mathrm{FN}}$(3)
Specificity (True Negative Rate)	$\mathrm{TNR}=\frac{\mathrm{TN}}{\mathrm{TN}+\mathrm{FP}}$(4)
Accuracy (ACC)	$\mathrm{ACC}=\frac{\mathrm{TP}+\mathrm{TN}}{\mathrm{TP}+\mathrm{TN}+\mathrm{FP}+\mathrm{FN}}$(5)
Precision (Positive Predictive Value)	$\mathrm{PPV}=\frac{\mathrm{TP}}{\mathrm{TP}+\mathrm{FP}}$(6)
Matthews Correlation Coefficient (MCC)	$\mathrm{MCC}=\frac{\mathrm{TP}\times \mathrm{TN}-\mathrm{FP}\times \mathrm{FN}}{\sqrt{\left(\mathrm{TP}+\mathrm{FP})(\mathrm{TP}+\mathrm{FN})(\mathrm{TN}+\mathrm{FP})(\mathrm{TN}+\mathrm{FN}\right)}}$(7)

where, TP are true positives (high effect treatments correctly classified as “high effect”), TN are true negatives (low effect treatments correctly classified as “low effect”), FP are false positives (low effect treatments incorrectly classified as “high effect”) and FN are false negatives (high effect treatments incorrectly classified as “low effect”).

The above mentioned metrics are also calculated for the test set treatments and are presented in the results. The test set treatments are original values, selected in a similar manner to the selection of the blind set treatments. However, we consider that the true performance of the model (e.g., in a real-case scenario) is reflected in the blind set metrics, as the test set participates in the selection of the final model.

Finally, the Y-randomisation test [77], [78] is conducted to verify that the selected model’s accuracy is not due to chance correlation. Within the Y-randomisation method, the training set’s endpoint values are randomly shuffled between treatments. All modelling steps are repeated several times using the original values of the independent descriptors and shuffled values for the endpoint variable. Then, if the original model is robust and reliable, it is anticipated that the randomised models perform poorly when the Y-randomised models are applied to the test or blind set treatments.

### 3.6 Applicability domain

The reductionist nature of nanoinformatics ML models leads to limitations in terms of chemical structures, physicochemical properties and mechanisms within a response space [79], therefore it is unrealistic to expect -even for the most powerful nanoQSAR/read-across models- to produce reliable predictions for all possible input data [80]. In general, extrapolated predictions (inference outside of the known training NPs hyperspace) are considered less accurate than interpolated ones (inference into the hyperspace defined by the training NPs). The definition of the applicability domain (AD) -also required by the OECD principles for QSAR validation (principle number 3)- determines the area of reliable predictions based on the training NPs and it is necessary for describing the limitations of the model. There are different methodologies in order to assess the AD of QSAR models, as described by Gadaleta *et al*. [81]. However, there is no one-size-fits-all AD approach agreed upon by every stakeholder including modellers, industry, and regulators [80].

To assess the AD of the models we propose a comprehensive approach where different AD assessment methods are combined and supplement each other, to enhance the confidence of stakeholders in the produced predictions. Similar approaches are proposed in the literature e.g., Dimitrov *et al*. [82] proposed a stepwise approach to assess the AD of QSAR models evaluating the range of variation of the descriptors, the compounds structural similarity, as well as a mechanistic and a metabolic check.

In this work, three approaches are integrated in the AD scheme: the bounding box method, the leverage method, and the local similarity method.

• In the bounding box (or range-based) method the interpolation space is considered the hyper-box defined by the range of minimum and maximum selected descriptor values. An untested NP is outside the AD, if at least one of its descriptor values is out of the range of the limits of the corresponding descriptor defined by the training NPs [83], [84]. This AD approach is simple and straightforward and does not comprise any adjustable hyperparameters [84]. Nonetheless, this method is reliable in cases where the descriptors follow a normal distribution. In addition, the hyper-box defined by this approach may consist of “hollow” data regions, where the interpolation relationship is not proven, and thus the predictions for untested NPs in these regions may be considered unreliable[79]. For categorical descriptors (e.g., in the case of the type of coating on the NPs) the corresponding range is the list of available labels of the training set. If the query NP’s categorical descriptor label is not included to the training descriptor labels list, the NP is outside the AD limits.
• In the leverage method, the leverage values h which are the diagonal elements of the Hat matrix [85], [86] (Eq. 8), reflect the similarity of the validation or untested samples to the training set (distance from the training set’s centroid [85]) based on the descriptor values used in the model development. The limits of the AD are determined by the threshold leverage value $h^{*}$ (Eq. 9). The prediction for a validation or untested NP is considered reliable if $h<h^{*}$.
  (8) $$H=X{\left(X^{T}X\right)}^{-1}X^{T}$$
  (9) $$h^{*}=3\times \frac{k}{N}\;$$
  where, X, is the table containing the training data, k, is the number of descriptors used in the specific model [85], and N, is the number of NPs in the training set.
  There are no strict rules for the definition of the $h^{*}$value; e.g., in Eq. 9 the *k* parameter is often defined as the number of descriptors used in the model plus one [79], [80], [81]. Furthermore, in this definition the size of the training set affects the threshold leverage value and, in fact, a smaller training set leads to an increased AD boundary. This is contradictory to the notion that larger and more diverse sets lead to extended ADs, and was explored by Gajewicz [80]: As the number of training NPs (N) decreases, the ratio k/N increases proportionately. Consequently, the threshold leverage value $h^{*}$also increases, resulting in a wider range of leverage values considered acceptable for untested NPs, even if these NPs are not truly similar to the training NPs. In addition, the use of the leverage method assumes that the data is normally distributed, or if the dataset is sufficiently large (more than 30 NPs) it can be weakly assumed that the source population is normally distributed [80] even if the data of the set are not normally distributed according to the central limit theorem. Nonetheless, nanoinformatics datasets are usually smaller than cheminformatics and bioinformatics sets [34] and sometimes are limited to less than 15 training NPs [80], therefore the assumption of a normal distribution cannot be established. In this work, the training NPs of the dataset are more than 57 samples, and thus we can assume that they come from a normally distributed population.
• To address the above mentioned issues with the bounding box and the leverage approaches, we propose a complementary AD approach based on the similarity of the closest training NPs to the query NP. In detail, this approach starts by applying the *k*NN methodology to the query NPs to assess the NP’s local region in the hyperspace. For each query NP the *k* closest training NPs are selected based on the Euclidean distances between them, calculated considering the selected descriptors. Next, the cosine similarity between the query NP and each of the *k* training NPs is calculated and it is compared to a predefined threshold ($\mathrm{si}m_{k}$). If the similarity value of at least one of the *k* training NPs is below the threshold, the query NP is out of the AD limits and the prediction for this NP is considered unreliable. Users should select the number of *k* neighbours and the similarity threshold value. In this work, these hyperparameters are adjusted as *k* = 5 and $\mathrm{si}m_{k}$ = 0.8. Overall, the *k*NN methodology combined with the cosine similarity calculation, provides a straightforward manner to determine whether an NP is within the AD limits or not even with small datasets, as it assesses the local space of a query NP (e.g., lower similarity values of the *k* neighbours indicate that the query NP region is hollow and vice versa). The use of the $\mathrm{si}m_{k}$ limit is an intuitive and meaningful measure to tune the desired trade-off between the models’ extent of use and the reliability of their predictions. Therefore, this method addresses the weaknesses of the previous approaches (hollow regions, uncertainties regarding the definition of the threshold values, small datasets), thereby increasing confidence in the predictions.

For any query NP it is possible to assess its reliability based on the AD using a scoring system inspired by the work of Roy *et al*.[87]. In this scheme the outcome of each of the three described AD methods is combined in a weighted score to assess the overall reliability of the prediction (Eq. 10).

(10) $$\mathrm{scor}e_{i}=w_{\mathrm{bb}}∙AD_{\mathrm{bb},i}+w_{\mathrm{lev}}∙AD_{\mathrm{lev},i}+w_{\mathrm{sim}}∙AD_{\mathrm{sim},i}$$

where, $\mathrm{scor}e_{i}$, is the combined reliability score of the $i^{\mathrm{th}}$ query NP, $w_{\mathrm{bb}}$, $w_{\mathrm{lev}}$, and $w_{\mathrm{sim}}$ are the weighting factors of the bounding box, leverage and similarity AD methods respectively, and $AD_{\mathrm{bb},i}$, $AD_{\mathrm{lev},i}$, and $AD_{\mathrm{sim},i}$ are binary variables indicating whether the $i^{\mathrm{th}}$ query NP is inside (value of 1) or outside (value of 0) the AD limits of the model according to the three AD methods.

The weighting factors can be user-defined parameters permitting them to tune the influence of each method on the final reliability outcome, or they can be defined as follows: As the similarity approach is more susceptible to the local space of the query NP with regards to the training NPs than the bounding box and the leverage methods, we propose that the $w_{\mathrm{sim}}$ is larger than $w_{\mathrm{bb}}$ and $w_{\mathrm{lev}}$. The proposed default values for each weighting factor are: $w_{\mathrm{bb}}=0.2$, $w_{\mathrm{lev}}=0.3$, and $w_{\mathrm{sim}}=0.5$.

Finally, the overall reliability of the prediction is proposed to be defined as follows:

$$\mathrm{scor}e_{i}<0.5\to \mathrm{Poor}\;\mathrm{reliability}$$

$$\mathrm{scor}e_{i}=0.5\to \mathrm{Moderate}\;\mathrm{reliability}\;$$

$$\mathrm{scor}e_{i}>0.5\to \mathrm{Good}\;\mathrm{reliability}$$

At this point we have to re-emphasise that the models are built using data for Ag, TiO_2_ and CuO NPs, therefore if they are used to predict the behaviour of other types of NPs (extrapolation), the differences between them and the training NPs should be considered. In addition, the models are developed considering the descriptors with high information gain regarding the classification endpoint (adverse effects towards the HepaRG cell line). Thus, generalising for other toxicity endpoints using the same descriptors and consequently the same AD limits, should be performed with caution.

## 4 Results

The goal of this study was to develop a predictive model for the *in silico* assessment of the toxicity of Ag, TiO_2_, and CuO NPs towards the HepaRG cell line using computationally derived descriptors encoding the structural and the physicochemical properties of the NPs. The KNIME (Konstanz Information Miner) Analytics Platform [68] was used to perform data analysis (including the synthetic data generation and filtering), modelling and validation, as well as defining the applicability domain. For this purpose, different extensions were integrated into the KNIME workflow such as the Enalos+ nodes [78], the R programming language [88], the Palladian [89] nodes and the AutoML component [76]. The AutoML component was customised to incorporate the synthetic data generation, the filtering, and the variable selection steps of the analysis prior to modelling. Later, the deployment of the model as a user-friendly application was made via the Isalos Analytics Platform [90] which permits deployment and sharing of ML models as web-services for straightforward access by the broader community, via the Enalos Cloud Platform. A summary of the modelling results is available in the supplementary file, presented in a QMRF report, as per regulatory validation requirements.

Initially, 30% of the original toxicity dose-response dataset and the NP atomistic descriptors was randomly chosen and set aside from the model development process. This subset, referred to as the blind set, was used later for validation. The rest of the data was filtered to remove descriptors with missing values and was split again randomly into training and test sets in a ratio of 75:25. A stratified sampling technique was applied to ensure that the class distribution (“low effect”/ “high effect”) in both test and blind datasets is representative of the original data.

Considering the class imbalance (66% “low effect” vs. 33% “high effect” treatments) in the training set, the minority class (“high effect”) was oversampled to ensure that the number of treatments for each endpoint class is approximately equal, by employing two oversampling methodologies: the SMOTE and the ADASYN using *k* = 5 neighbours in both cases. It should be noted that descriptors expressing ratios or differences between core and surface atoms were filtered out before oversampling and recalculated afterward to preserve their original properties.

In order to select the most appropriate -in terms of compatibility to the original set- between the synthetic training sets produced with the two methodologies (SMOTE and ADASYN) the utility of the synthetic data was assessed. For the data oversampled by either method, original and synthetic training treatments were marked with the indicators 0 and 1, respectively. Later the data were filtered using a low-variance filter (threshold of 0.2) and a correlation filter (threshold of 0.9 in the Spearman’s rank correlation coefficient) and the remaining descriptor values were normalised according to the z-score method. Subsequently, a logistic regression model was developed using the LOO cross-validation technique to predict the subset class (0 or 1), and the pMSE value was calculated according to Eq. 1. The pMSE values for the SMOTE and ADASYN oversampled data is presented in Table 5. As also discussed in the §Methods, pMSE values closer to zero indicate greater similarity between the original and oversampled sets. For this reason, even if the two methodologies present low pMSE values, the data produced with the ADASYN methodology were considered the most appropriate and were finally used in the rest of the analysis.

**Table 5:** pMSE values for the oversampled training sets using the SMOTE and ADASYN oversampling methodologies.

	pMSE
SMOTE	0.109
ADASYN	0.091

For the oversampled set following the ADASYN method, the KL divergence and the Wasserstein distance metrics were calculated, as well. The KL divergence was calculated using the KNIME Kullback–Leibler divergence component [91] for all the descriptors and their values ranged from 0.001 to 0.065. The Wasserstein distances were calculated for all descriptors in Python using the SciPy module [92] and their values varied from 0.009 to 0.044. In both cases, the low metrics values suggest that there is a high degree of similarity between the original and the oversampled datasets, indicating a minimal loss of fidelity in the distribution of the descriptors due to oversampling. Thus, the ADASYN methodology effectively generated the synthetic NP treatments, preserving in this way the underlying structure of the original data and consequently, ensuring the development of reliable ML models.

After the oversampling of the underrepresented class, training data (75 NP treatments) were fed into a low variance filter and to a Spearman’s rank correlation coefficient filter, to remove non-essential descriptors. For the remaining descriptors, the information gain was calculated [89] with the purpose to select the most significant descriptors (information gain score greater than zero) for modelling (see Table 6). Once the filtering was complete, the training set was examined to identify and remove any duplicate treatments that may have arisen from the descriptor filtering steps, because possible repeated treatments may influence the result of the ML models [74]. The last preprocessing step was the training data normalisation using the z-score methodology.

**Table 6:** List of selected descriptors using the information gain method.

Selected descriptors	Information gain score
Concentration of NPs in μg/ML	0.482
AD45: The average difference of the CNP (3Ang) between core and shell atoms	0.145
AD27: The average difference of the coordination parameter (5Ang) between core and shell atoms	0.145
AD22: The average difference of the coordination parameter (4Ang) between core and shell atoms	0.145
AD17: The average difference of the coordination parameter (3Ang) between core and shell atoms	0.145
AD16: The average coordination parameter (3Ang) of the shell atoms	0.169
AD14: The average coordination parameter (3Ang) of all atoms	0.145
AD9: The average coordination parameter of all atoms	0.145
AD7: The average difference of the potential energy between core and shell atoms in eV	0.150
AD3: Log10 of all atoms in the surface	0.145
AD1: Log10 of all atoms in the NP	0.145

The seven models were trained using five-fold cross validation and the hyperparameters were iteratively optimised as presented in Table 7. Next, the optimised models were applied to the test set treatments (in total 20 NP treatments), which were previously normalised according to the training set normalisation parameters, and the accuracy statistics metrics were calculated as presented in Table 8. Considering all the statistical metrics of the application of the different ML models on the test set, the random forest model was selected to proceed with the rest of the analysis.

**Table 7:** Optimised hyperparameters of the ML methodologies in the autoML scheme after five-fold cross validation.

ML methodologies	Optimised hyperparameters
Gradient boosted trees	nrModels = 50
Naïve Bayes	threshold = 0.0065
Logistic regression	stepSize = 0.01
Decision tree	minNumberRecordsperNode = 16
Random forest	maxLevels = 4, minNodesize = 20, nrModels = 200
Neural network	hiddenlayer = 1, nrhiddenneurons = 15
XGBoost trees	eta = 0.2, Max_depth = 5

**Table 8:** Accuracy statistics on the test set of the assessed methodologies in the autoML scheme.

ML methodologies	Accuracy	Cohen’s kappa	Recall	Precision	Specificity	MCC
Gradient Boosted Trees	0.90	0.78	0.86	0.86	0.92	0.78
Naive Bayes	0.50	0.17	1.00	0.41	0.23	0.31
Logistic Regression	0.85	0.66	0.71	0.83	0.92	0.66
Decision Tree	0.80	0.61	1.00	0.64	0.69	0.66
Random Forest	0.95	0.89	1.00	0.88	0.92	0.90
Neural Network	0.85	0.66	0.71	0.83	0.92	0.66
XGBoost Trees	0.90	0.78	0.86	0.86	0.92	0.78

**Fig. 2:**
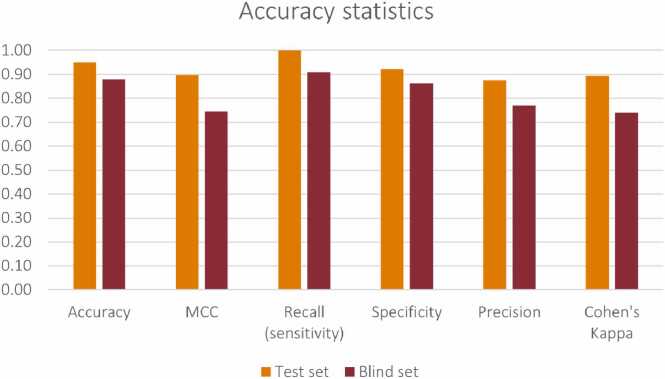
Accuracy statistics of the random forest model when applied on the test and blind datasets.

The random forest ML model was applied to the blind set NP treatments (33 treatments) that were previously normalised according to the training set normalisation parameters. The performance of the developed model was encoded in different statistical metrics as presented in Table 9 and visualised in Fig. 2. The values of the statistical metrics denote a good agreement between actual and predicted values, demonstrating that the model produces reliable predictions in a real-case scenario. The results of the Y-randomisation test performed 10 times are presented in Table 10 in terms of accuracy and MCC values, when the shuffled-endpoint random forest models are applied on the test set. This test confirmed that the random forest model is robust and that the predictions are not a coincidental outcome.

**Table 9:** Accuracy statistics of the random forest model when applied on the blind dataset.

Metric	Value
Accuracy	0.88
Cohen’s Kappa	0.74
Recall (sensitivity)	0.91
Precision	0.77
Specificity	0.86
MCC	0.75

**Table 10:** Results of the Y-randomisation test, expressed as accuracy and MCC, when the shuffled-endpoint random forest models were applied on the test set.

Randomisation	Accuracy	MCC
1	0.25	-0.42
2	0.45	-0.10
3	0.35	-0.21
4	0.65	0.31
5	0.60	0.12
6	0.50	0.10
7	0.60	0.32
8	0.40	-0.24
9	0.55	0.10
10	0.60	0.18

Finally, the AD was defined as previously described: According to the bounding-box approach, we evaluated whether the descriptor values of the blind set treatments fell within the range defined by the minimum and maximum descriptor values of the training set. Since the blind set treatments were within these ranges, they are inside the AD, as well. The AD threshold leverage value was calculated equal to 0.440 and all blind set NP treatments had leverage values in the range of 0.098–0.425 except one, thus according to this approach the blind set NP treatments were again located inside the AD, except the one treatment with greater leverage value (0.490) than the threshold. When applying the similarity approach, the local space (5 closest neighbours) of six NP treatments in the blind set did not meet the minimum similarity requirement of 0.8 (they are located in a “hollow” region of the bounding-box), thus these treatments are located outside the AD. The overall reliability of the predictions of the blind set was assessed through the proposed score value (Eq. 10) and the results are presented in the supporting information file of this publication.

### 4.1 Descriptors space

During application of the SSbD framework to the development of novel NPs, it is essential to assess how the NP properties and physicochemical characteristics influence their biological effects and toxicity. Thus, once predictive ML models for NPs toxicity are developed and validated, the key variables involved in these models and thus driving toxicity should be interpreted, to determine whether adjustments to the NPs properties could reduce their toxicity. In this study, the assessment of the information encoded within the selected computational descriptors gives insight into their impact to the NPs toxicity, prior to the NPs actual synthesis since no experimental data is needed to make use of the model, allowing a fast virtual screening of large sets of NP treatments i.e., NP sizes, shapes, crystal phase (for TiO_2_ NPs) etc., particularly for the three NP compositions included in the ML model (i.e., Ag, TiO_2_ and CuO).

The variable influencing the toxicity endpoint most is the concentration of NPs to which the HepaRG cells were exposed (see information gain score in Table 6). The selection of concentration is rather obvious, considering that the data used for modelling are derived from dose-response experiments and thus, concentration values outline the experimental conditions. Nonetheless, the use of the concentration itself as the only training descriptor is not sufficient to produce a reliable ML model, as the concentration values are repeated across NP treatments, and thus may introduce bias into the model[74]. The use of the atomistic descriptors contributes to the generation of more reliable predictions, by introducing into the model information on the NP core material. More importantly, the use of atomistic computations allows the transition from the microscopic to the macroscopic level in the assessment of the influence of NP properties on the toxicity they induce in cells.

In the calculation of the atomistic descriptors a threshold of 4 Å was defined to separate core and shell (surface) atoms in spherical particles which leads to surface thickness of 4 Å. Concerning the ellipsoid particles the core and shell atoms are defined by subtracting 4 Å from their axes semilengths to be compatible with the sphere definition (e.g., equal to the sphere surface thickness in the limit of equal ellipsoid axes) or rod definition (e.g., surface thickness of 4 Å). These thresholds were selected from the assessment of the average potential energy per atom of various TiO_2_, CuO, and Ag NPs, which stabilises within 4 Å from the NPs’ surface. This threshold of 4 Å also coincides with the predicted optimal shell depth (https://nanogen.me/shell-depth) proposed by Burk *et al.*[93] and Tämm *et al*.[94] for TiO_2_-Anatase (3 nm and 5 nm diameter NPs) and CuO (5 nm and 6 nm diameter NPs). Their predicted optimal shell depth for TiO_2_-Rutile (3 nm and 5 nm diameter NPs) is 5 Å while their prediction is limited to metal oxides. Using a threshold of 4 Å for all cases enables a fair comparison of descriptors across different NPs, including those with varying chemical compositions.

**Fig. 3:**
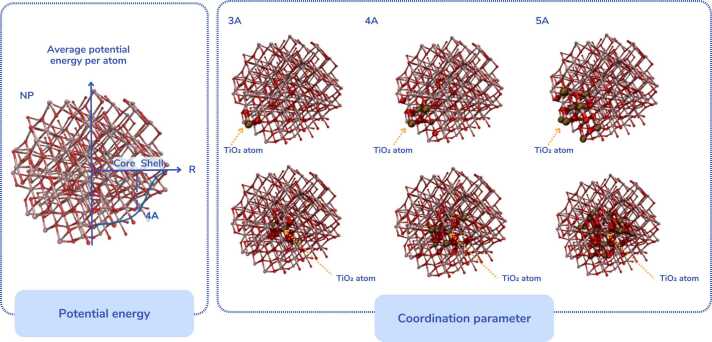
Left: qualitative sketch of a TiO_2_ NP (of 5 nm diameter) depicting the average potential energy per atom. Ti atoms are depicted in pink colour and O atoms are depicted in red colour. Right: the number of neighbouring atoms for a Ti atom in the shell (upper NPs) and in the core (lower NPs) at 3, 4, and 5 Å from the Ti atom. The neighbouring atoms are highlighted in the graphic with increased thickness.

The selected atomistic descriptors include the difference of the average potential energy between the core and the shell atoms (Fig. 3). The average potential energy group of descriptors expresses the stability of the particles (e.g., lower average potential energy values of the atoms correspond to more stable structures), while the common neighbour parameter (CNP) is a useful indicator of the local crystal structure around an atom and can be used to determine whether the atom is located in a perfect lattice, at a surface, or is part of a local defect[95]. The average coordination number (Fig. 3) expresses the number of neighbouring atoms for a single atom. Every atom that is away less than a pre-defined distance (see the neighbours of Ti atoms at 3 Å, 4 Å, and 5 Å distances in Fig. 3) is considered a neighbouring atom. Descriptors defined as differences of the descriptors of the core and surface atoms are very informative of NPs reactivity: if there is a significant difference in the average number of neighbouring atoms between core and shell (surface) atoms, the surface of the NP is expected to be highly reactive as there will be a significant number of unterminated bonds which could be recreated after the reaction of the surface atoms with external atoms such as those present in the surrounding medium (Fig. 3, see differences between the top and bottom NP representations where the neighbours of a shell and a core Ti atom are depicted). Therefore, higher values of these descriptors indicate highly reactive NPs. Finally, the log10 of all atoms of the NP is connected to the NP’s size and lower values of this descriptor indicate smaller NPs which in general are more toxic than larger particles at a constant mass, due to the larger number of smaller particles. Nonetheless, it should be noted that these descriptors are calculated for uncoated NPs in vacuum and thus, a direct relationship between the NPs descriptors-reactivity and their toxicity cannot be established at this point. Computational study of NP coatings, which is currently being developed, will further contribute to the understanding of the behaviour of NPs in biological media and thus, will elucidate the mechanisms that drive nanotoxicity.

A more comprehensive analysis of the selected descriptors can be found in the manual of the ASCOT web-application[53], [54].

## 5 Model availability via the Enalos SABYDOMA Cloud Platform

**Fig. 4:**
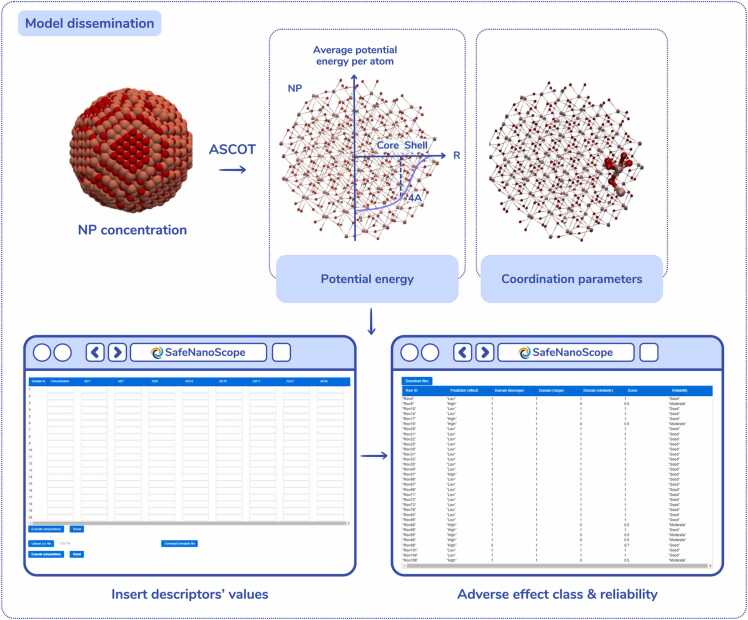
SafeNanoScope web application interface. Users can either input the required descriptors via the provided form or by uploading a CSV file with all the required properties. The output is a prediction of each NPs’ toxicity class and a comment on the reliability of the prediction based on the model’s domain of applicability.

The growing number of *in silico* approaches for the assessment of NPs’ adverse effects that have been developed recently highlights the urgent need for alternative NP screening methods and NAMs to support practical implementation of the SSbD framework. However, nanosafety researchers (such as experimentalists and regulatory specialists) who could directly benefit from the use of computational methodologies in their daily work may be discouraged from applying or assessing such models by the programming environments used (e.g., Python, R, etc.) due to a lack of a solid background knowledge in data science or programming, or due to time constraints that prevent them from learning to develop their own scripts and models. For this reason, the developed model is offered as a web application (the SafeNanoScope, www.enaloscloud.novamechanics.com/sabydoma/safenanoscope/) with an intuitive graphical user interface (GUI), specifically designed for non-informatics experts, simplifying the interaction with the model’s technical components. The SafeNanoScope web application (Fig. 4) is anticipated to play a significant role in future computer-aided NP design and quality control processes.

Users can employ the ASCOT tool (www.enaloscloud.novamechanics.com/sabydoma/ascot/), or another software of their choice, to provide the requested NP atomistic descriptors and the NPs concentration. In order to introduce the above information into the model there are two different options: users can either enter manually the necessary information using the form given in the application (advisable for small NP treatment sets), or they can import a file in CSV format containing the NP treatments data. After submitting the required information, predictions are produced and presented within seconds. The results include the predicted adverse effects’ class against the HepaRG cell line (“low” or “high”) and a warning on the prediction reliability according to the proposed AD methodology. Users can import multiple datasets with NP treatments and study their effects on the HepaRG cells, a critical step during the SSbD process.

## 6 Data availability via the nanoPharos database

The availability and quality of data are paramount for the successful development of predictive models, including nanoQSAR and read-across methodologies which leverage data-driven ML techniques. These methods are inherently reliant on robust and comprehensive datasets to effectively predict the behaviour and interactions of NPs. However, a significant challenge within the nanosafety community is the scarcity and inaccessibility of such data[34]. Addressing this challenge is crucial, and a pivotal strategy in this regard is the FAIRification of data and metadata, aligning with the principles of Findability, Accessibility, Interoperability, and Reusability[96]. The nanoPharos database has been developed as a repository that not only offers data in a format ready for modelling but also embodies the ethos of FAIR. The inclusion of computationally derived data, encompassing simulations of NPs across a spectrum of complexity levels into nanoPharos, both as enrichment of existing toxicity datasets or as standalone data, enhances the breadth of data available for modelling and thus the research scope.

In the context of the WorldFAIR project[97], [98], which aims to promote global cooperation on FAIR data policy and practice, the nanoPharos database represents a significant advance towards a universally accessible and reusable data repository to support NPs safety assessment. The nanoPharos database, enables straightforward sharing of data and serves as a model platform, demonstrating how nanosafety data can be curated and managed in accordance with international standards. Moreover, by including a broad range of computationally derived data and detailed NP characterisation, the nanoPharos database contributes to enriching the global data pool, and by making the datasets open and FAIR is playing a pivotal role in this shift towards more open, collaborative, and efficient research methodologies. The enriched dataset used in the development of the presented ML model, SafeNanoScope, is thus made available through the nanoPharos database in a ready-for-modelling format (https://db.nanopharos.eu/Queries/Datasets.zul?datasetID=16).

## 7 Conclusions

The development of reliable and accurate computational strategies, grounded in the latest research and technological advances, is key to accelerating the hazard and risk assessment of NPs and facilitating the development of SSbD NPs. These computational strategies can serve as alternatives to traditional regulatory assessment methods applied to substances as they come to market, by enabling the assessment of NPs properties before they are used in commercial products or even before they are synthesised. The creation of specific nano-descriptors is a critical step in this process, as it allows the *in silico* exploration of the connections between the NPs’ structure, their properties and/or their biological effects.

In this study, a ML model for the prediction of the adverse effects of Ag, TiO_2_, and CuO NPs of different sizes and shapes is presented. The model was based on NPs’ dose-response toxicity data enriched with computationally derived atomistic NP descriptors, which were used as the independent variables for the prediction of the NPs’ toxicity class (low or high toxicity). The high toxicity data was oversampled to balance the representation of classes in the set, by generating synthetic data using two methodologies, SMOTE and ADASYN. The quality of the synthetic data was also assessed to ensure the compatibility of the synthetic data with the original data. The model presented here was produced from an autoML scheme, where the best-performing model between 7 optimised models was selected, i.e., the random forest ML model. Finally, a novel AD scheme is applied to the data to define the area of the reliable predictions by assessing three different criteria of similarity between the query and the training data.

This cutting-edge methodology contributes to the study of the adverse effects of NPs demonstrating the potential of utilising knowledge of the system structure and composition as the basis for prediction. This eliminates the need for experimental data to assess NP safety, enabling risk and hazard assessments to be performed before the actual NP synthesis and production, as is already applied in the drug discovery pipeline. Consequently, large sets of theoretically constructed NPs can be virtually screened to rapidly evaluate their desired properties (e.g., enhanced mechanical/electronic/targeting properties and reduced toxicity) and therefore it is possible to prioritise promising candidates for synthesis and further evaluation, leading to the production of NPs that are SSbD. Finally, the dissemination of the NPs’ digital reconstruction code and the ML model as user-friendly tools via the Enalos Cloud Platform with full documentation including a QMRF report and training guide, and their interconnection under an IATA framework, will foster their adoption by stakeholders in regulatory and industrial sectors. Future work should focus on the modelling of the NPs’ surface including the calculation of computational descriptors for the NPs’ coating, considering that the NPs surface properties influence NP behaviour and interactions with biomolecules, membranes, and organisms.

## CRediT authorship contribution statement

**Dimitra-Danai Varsou:** Conceptualization, Methodology, Formal analysis, Data Curation, Writing - Original Draft, Writing - Review & Editing, Visualization. **Panagiotis D. Kolokathis:** Resources, Writing - Review & Editing. **Maria Antoniou:** Data Curation. **Nikolaos K. Sidiropoulos:** Software. **Andreas Tsoumanis:** Software. **Anastasios G. Papadiamantis:** Data Curation. **Georgia Melagraki:** Writing - Review & Editing. **Iseult Lynch:** Writing - Review & Editing. **Antreas Afantitis:** Conceptualization, Writing - Review & Editing, Supervision, Funding acquisition.

## Declaration of Competing Interest

DDV, PK, MA, NKK, AT, AGA and AA are affiliated with NovaMechanics, a cheminformatics and materials informatics company
