# Supplementary material for: In silico assessment of nanoparticle toxicity powered by the Enalos Cloud Platform: Integrating automated machine learning and synthetic data for enhanced nanosafety evaluation
Source: Comput Struct Biotechnol J. 2024 Mar 30;25:47–60. doi: 10.1016/j.csbj.2024.03.020 (PMC11026727; doi:10.1016/j.csbj.2024.03.020)
Supplement: Supplementary file 1 — Supplementary material. [file mmc1.pdf]

In Silico Assessment of Nanoparticle Toxicity Powered by the Enalos Cloud Platform: Integrating Automated Machine Learning and Synthetic Data for Enhanced Nanosafety Evaluation

Dimitra-Danai Varsou[1,2,†], Panagiotis D. Kolokathis[1,2], Maria Antoniou[3], Nikolaos K. Sidiropoulos[1,2], Andreas Tsoumanis[2,3], Anastasios G. Papadiamantis[2,3,4], Georgia Melagraki[5], Iseult Lynch[2,4], Antreas Afantitis[1,2,3,†]

[1] NovaMechanics MIKE, Piraeus, 18545, Greece

[2] Entelos Institute, Larnaca, 6059, Cyprus

[3] NovaMechanics Ltd, Nicosia 1070, Cyprus

[4] School of Geography, Earth and Environmental Sciences, University of Birmingham, B15 2TT, Birmingham, UK

[5] Division of Physical Sciences and Applications, Hellenic Military Academy, Vari 16672, Greece

[†]Corresponding author

Supporting information

Table 1: List of atomistic descriptors calculated by the ASCOT method.

Symbol	Description
AD1	Log10 of all atoms in the NP
AD2	Log10 of all atoms in the core
AD3	Log10 of all atoms in the surface
AD4	The average potential energy of all atoms in eV
AD5	The average potential energy of the core atoms in eV
AD6	The average potential energy of the shell atoms in eV

AD7	The average difference of the potential energy between core and shell atoms in eV
AD8	The average ratio of the potential energy between core and shell atoms in eV
AD9	The average coordination parameter of all atoms
AD10	The average coordination parameter of the core atoms
AD11	The average coordination parameter of the shell atoms
AD12	The average difference of the coordination parameter between core and shell atoms
AD13	The average ratio of the coordination parameter between core and shell atoms
AD14	The average coordination parameter (3Ang) of all atoms
AD15	The average coordination parameter (3Ang) of the core atoms
AD16	The average coordination parameter (3Ang) of the shell atoms
AD17	The average difference of the coordination parameter (3Ang) between core and shell atoms
AD18	The average ratio of the coordination parameter (3Ang) between core and shell atoms
AD19	The average coordination parameter (4Ang) of all atoms
AD20	The average coordination parameter (4Ang) of the core atoms
AD21	The average coordination parameter (4Ang) of the shell atoms
AD22	The average difference of the coordination parameter (4Ang) between core and shell atoms
AD23	The average ratio of the coordination parameter (4Ang) between core and shell atoms
AD24	The average coordination parameter (5Ang) of all atoms
AD25	The average coordination parameter (5Ang) of the core atoms
AD26	The average coordination parameter (5Ang) of the shell atoms
AD27	The average difference of the coordination parameter (5Ang) between core and shell atoms
AD28	The average ratio of the coordination parameter (5Ang) between core and shell atoms
AD29	The average coordination parameter (6Ang) of all atoms
AD30	The average coordination parameter (6Ang) of the core atoms
AD31	The average coordination parameter (6Ang) of the shell atoms
AD32	The average difference of the coordination parameter (6Ang) between core and shell atoms
AD33	The average ratio of the coordination parameter (6Ang) between core and shell atoms

AD34	The diameter of the NP in A
AD35	The surface area of the NP in A^2
AD36	The volume of the NP in A^3
AD37	Lattice energy of NP in eV
AD38	Lattice energy of bulk material - Lattice energy of NP in eV
AD39	Lattice energy of NP divided by the NP surface in eV/A^2
AD40	Lattice energy of NP divided by the NP volume in eV/A^3
AD41	Lattice energy of bulk material / Lattice energy of NP
AD42	The average CNP (3Ang) of all atoms
AD43	The average CNP (3Ang) of the core atoms
AD44	The average CNP (3Ang) of the shell atoms
AD45	The average difference of the CNP (3Ang) between core and shell atoms
AD46	The average ratio of the CNP (3Ang) between core and shell atoms
AD47	The average first hex parameter of all atoms
AD48	The average first hex parameter of the core atoms
AD49	The average first hex parameter of the shell atoms
AD50	The average second hex parameter of all atoms
AD51	The average second hex parameter of the core atoms
AD52	The average second hex parameter of the shell atoms

Table 2: Predictions of the blind set and their reliability according to the applicability domain approach.

NanoMILE ID	Metal core	Shape group	Size in ASCOT [nm]	Concentration [μg/mL]	Overall toxicity	Prediction (Overall toxicity)	Applicability domain (leverage)	Applicability domain (range)	Applicability domain (similarity)	Score	Reliability
NP00214	Ag	Spherical	21.097	7.81	High effect	Low effect	1	1	1	1	Good
NP00214	Ag	Spherical	21.097	250	High effect	High effect	1	1	0	0.5	Moderate
NP00432	Ag	Spherical	19	0.49	Low effect	Low effect	1	1	1	1	Good
NP00432	Ag	Spherical	19	7.81	Low effect	Low effect	1	1	1	1	Good

NP00432	Ag	Spherical	19	62.5	High effect	High effect	1	1	1	1	Good
NP00432	Ag	Spherical	19	250	High effect	High effect	1	1	0	0.5	Moderate
NP00255	TiO_2	Spherical	9.95	0.49	Low effect	Low effect	1	1	1	1	Good
NP00255	TiO_2	Spherical	9.95	0.98	Low effect	Low effect	1	1	1	1	Good
NP00255	TiO_2	Spherical	9.95	1.95	Low effect	Low effect	1	1	1	1	Good
NP00255	TiO_2	Spherical	9.95	15.63	Low effect	High effect	1	1	1	1	Good
NP00256	TiO_2	Square	11.65	0.49	Low effect	Low effect	1	1	1	1	Good
NP00256	TiO_2	Square	11.65	0.98	Low effect	Low effect	1	1	1	1	Good
NP00256	TiO_2	Square	11.65	3.91	Low effect	Low effect	1	1	1	1	Good
NP00256	TiO_2	Square	11.65	15.63	Low effect	High effect	1	1	1	1	Good
NP00257	TiO_2	Square	13.32	0.49	Low effect	Low effect	1	1	1	1	Good
NP00258	TiO_2	Square	13.24	62.5	High effect	High effect	1	1	1	1	Good
NP00259	TiO_2	Rods	40.7/25.3	31.25	Low effect	Low effect	1	1	1	1	Good
NP00259	TiO_2	Rods	40.7/25.3	62.5	Low effect	Low effect	1	1	1	1	Good
NP00259	TiO_2	Rods	40.7/25.3	250	Low effect	Low effect	1	1	1	1	Good
NP00260	TiO_2	Rods	42.5/23.2	0.98	Low effect	Low effect	1	1	1	1	Good
NP00260	TiO_2	Rods	42.5/23.2	1.95	Low effect	Low effect	1	1	1	1	Good
NP00260	TiO_2	Rods	42.5/23.2	3.91	Low effect	Low effect	1	1	1	1	Good
NP00260	TiO_2	Rods	42.5/23.2	250	Low effect	Low effect	1	1	1	1	Good
NP00441	TiO_2	Spherical	9.5	3.91	Low effect	Low effect	1	1	1	1	Good
NP00441	TiO_2	Spherical	9.5	15.63	Low effect	High effect	1	1	1	1	Good
NP00441	TiO_2	Spherical	9.5	125	High effect	High effect	1	1	1	1	Good
NP00441	TiO_2	Spherical	9.5	250	High effect	High effect	1	1	1	1	Good
NP00458	CuO	Spherical	12.144	15.63	High effect	High effect	1	1	0	0.5	Moderate
NP00458	CuO	Spherical	12.144	125	High effect	High effect	1	1	0	0.5	Moderate
NP00458	CuO	Spherical	12.144	250	High effect	High effect	0	1	0	0.2	Poor
NP00456	CuO	Spherical	5.95	0.98	Low effect	Low effect	1	1	1	1	Good
NP00456	CuO	Spherical	5.95	7.81	Low effect	Low effect	1	1	1	1	Good
NP00456	CuO	Spherical	5.95	125	High effect	High effect	1	1	0	0.5	Moderate
