# Supplementary material for: In silico assessment of nanoparticle toxicity powered by the Enalos Cloud Platform: Integrating automated machine learning and synthetic data for enhanced nanosafety evaluation
Source: Comput Struct Biotechnol J. 2024 Mar 30;25:47–60. doi: 10.1016/j.csbj.2024.03.020 (PMC11026727; doi:10.1016/j.csbj.2024.03.020)
Supplement: Supplementary file 2 — Supplementary material. [file mmc2.pdf]

(Q)SAR model reporting format (QMRF)

	Element	Explanation
**1.**	**QSAR identifier**	
1.1.	QSAR identifier (title)	SafeNanoScope: Ag-TiO_2-CuO safety assessment powered by Enalos SABYDOMA Cloud Platform
1.2	Other related models	Not applicable
1.3.	Software coding the model	https://www.enaloscloud.novamechanics.com/sabydoma/safenanoscope/
**2.**	**General information**	
2.0	Abstract	A nanoQSAR-type model for the prediction of the toxicity class of Ag, TiO_2 and CuO nanoparticles (NPs) based on their properties in atomistic level. More information can be found in the respective publication: Varsou *et al.* In Silico Assessment of Nanoparticle Toxicity Powered by the Enalos Cloud Platform: Integrating Automated Machine Learning and Synthetic Data for Enhanced Nanosafety Evaluation, *Computational and Structural Biotechnology Journal*, 2024.
2.1.	Date of QMRF	06 February 2024
2.2.	QMRF author(s) and contact details	Dimitra-Danai Varsou – varsou@novamechanics.com<br>Antreas Afantitis – afantitis@novamechanics.com
2.3.	Date of QMRF update(s)	Not applicable
2.4.	QMRF update(s)	Not applicable
2.5.	Model developer(s) and contact details	Dimitra-Danai Varsou – varsou@novamechanics.com
2.6.	Date of model development and/or publication	Date of model publication: February 2024.
2.7.	Reference(s) to main scientific papers and/or software package	Varsou *et al.* In Silico Assessment of Nanoparticle Toxicity Powered by the Enalos Cloud Platform: Integrating Automated Machine Learning and Synthetic Data for Enhanced Nanosafety Evaluation, *Computational and Structural Biotechnology Journal*, 2024.

2.8.	Availability of information about the model	The model is proprietary: the source code is confidential; however, the description of the modelling workflow is presented in the original research article, training and test sets are available as supplementary information of the original research article and the model is implemented as a public web service.
2.9.	Availability of another QMRF for exactly the same model	No
**3**	**Defining the endpoint - OECD Principle 1: “A DEFINED ENDPOINT"**	**PRINCIPLE 1: “A DEFINED ENDPOINT". ENDPOINT refers to any physicochemical, biological, or environmental property / activity / effect that can be measured and therefore modelled. The intent of PRINCIPLE 1 (a (Q)SAR should be associated with a defined endpoint) is to ensure clarity in the endpoint being predicted by a given model, since a given endpoint could be determined by different experimental protocols and under different experimental conditions. It is therefore important to identify the experimental system and test conditions that is being modelled by the (Q)SAR.**
3.1.	Species	Human hepatic cell line (HepaRG)
3.2.	Endpoint	The human hepatoma HepaRG cell line was treated with 89 NPs at 10 different concentrations, and 14 imaging endpoints were measured through a High Throughput Screening (HTS) – High Content Imaging (HCI) study to initially classify NP hazards and identify candidates for further toxicological assessment. The endpoints assessed included cell viability and mitochondrial health by measuring 9 features and the results of the HTS-HCI screening were normalised following the signal-to-noise ratio approach. A threshold of -3 for downward response and +3 for upward response was used, which corresponded to a 99% certainty that the cell behaviour was different from the untreated (negative) control value (cells treated only with medium).
3.3	Comment on endpoint	The normalised values were depicted in a colour-coded heatmap, which reflected the extend of difference of the behaviour from the untreated control (red and blue colours for decreased or increased response, respectively) or indicated similar behaviour to the untreated control (green colour).
3.4.	Endpoint units	The results of the 9 toxicity features were summarised into a single endpoint (“overall”) class as follows: NP treatments were classified as “Low effect” if they had a similar response to the negative controls (green labels) in at least 5 measured features (73 NP treatments). Otherwise, NP treatments were classified as “High effect” (red and/or blue label, 37 treatments).
3.5.	Dependent variable	Not applicable
3.6.	Experimental protocol	Information on the experimental protocols can be found in: Joossens, E., Macko, P., Palosaari, T. *et al.* A high throughput imaging database of toxicological effects of nanomaterials tested on HepaRG cells. Sci Data 6, 46 (2019). https://doi.org/10.1038/s41597-019-0053-2
3.7.	Endpoint data quality and variability	Information on the data quality and variability can be found in: Joossens, E., Macko, P., Palosaari, T. *et al.* A high throughput imaging database of toxicological effects of nanomaterials tested on HepaRG cells. Sci Data 6, 46 (2019). https://doi.org/10.1038/s41597-019-0053-2

**4**	**Defining the algorithm - OECD Principle 2 : “AN UNAMBIGUOUS ALGORITHM”**	**PRINCIPLE 2: “AN UNAMBIGUOUS ALGORITHM”. The (Q)SAR estimate of an endpoint is the result of applying an ALGORITHM to a set of structural parameters which describe the chemical structure. The intent of PRINCIPLE 2 (a (Q)SAR should be associated with an unambiguous algorithm) is to ensure transparency in the model algorithm that generates predictions of an endpoint from information on chemical structure and/or physicochemical properties. In this context, algorithm refers to any mathematical equation, decision rule or output approach.**
4.1.	Type of model	Type of model: Ensemble learner (Random Forest)
4.2.	Explicit algorithm	Random Forest: Random forests are supervised ensemble learning algorithms that utilize bagging (bootstrap aggregating) and feature randomness to construct a multitude of decision trees.
4.3.	Descriptors in the model	• Concentration of NPs in μg/mL<br>• The average difference of the common neighbour parameter, CNP, (local crystal structure in a diameter of 3Ang) between core and shell atoms [AD45*]<br>• The average difference of the coordination parameter (neighbouring atoms in a diameter of 5Ang) between core and shell atoms [AD27]<br>• The average difference of the coordination parameter (neighbouring atoms in a diameter of 4Ang) between core and shell atoms [AD22]<br>• The average difference of the coordination parameter (neighbouring atoms in a diameter of 3Ang) between core and shell atoms [AD17]<br>• The average coordination parameter (neighbouring atoms in a diameter of 3Ang) of the shell atoms [AD16]<br>• The average coordination parameter (neighbouring atoms in a diameter of 3Ang) of all atoms [AD14]<br>• The average coordination parameter of all atoms [AD9]<br>• The average difference of the potential energy between core and shell atoms in eV [AD7]<br>• Log10 of all atoms in the surface [AD3]<br>• Log10 of all atoms in the NP [AD1]<br>*This notation is consistent with the relevant publication.
4.4.	Descriptor selection	From the initial pool of descriptors (53 in total), 33 were filtered out using missing values, low variance and correlation filtering (see §6.6). The information gain of all remaining descriptors (20) is calculated and descriptors with zero information gain score are excluded from the modelling, as they are not considered critical for establishing a predictive relationship. Finally, 11 descriptors were selected (see §4.3).
4.5.	Algorithm and descriptor generation	Atomistic simulations. To perform the simulations and acquire the computational descriptors, the size, the shape, and the phase of the NPs were needed.
4.6.	Software name and version for descriptor generation	ASCOT: A Web Tool for the Digital Reconstruction of Energy Minimized Ag, CuO, and TiO_2 Spherical Nanoparticles and Calculation of their Atomistic Descriptors Powered by Enalos SABYDOMA Cloud Platform, https://www.enaloscloud.novamechanics.com/sabydoma/ascot/<br>For ellipsoid NPs see the NanoConstruct: Nanoparticle Construction Tool Powered by Enalos RiskGONE Cloud Platform, http://enaloscloud.novamechanics.com/riskgone/nanoconstruct/

4.7.	Chemicals/Descriptors ratio	75 training NP treatments (after oversampling)/ 11 selected descriptors. Optimised Random Forest parameters: maxLevels = 4, minNodesize = 20, nrModels = 200.
**5**	**Defining the applicability domain - OECD Principle 3: "A DEFINED DOMAIN OF APPLICABILITY"**	**PRINCIPLE 3: "A DEFINED DOMAIN OF APPLICABILITY". APPLICABILITY DOMAIN refers to the response and chemical structure space in which the model makes predictions with a given reliability. Ideally the applicability domain should express the structural, physicochemical and response space of the model. The CHEMICAL STRUCTURE (x variable) space can be expressed by information on physicochemical properties and/or structural fragments. The RESPONSE (y variable) can be any physicochemical, biological or environmental effect that is being predicted. According to PRINCIPLE 3 a (Q)SAR should be associated with a defined domain of applicability. Section 5 can be repeated (e.g., 5.a, 5.b, 5.c, etc) as many times as necessary if more than one method has been used to assess the applicability domain.**
5.1.	Description of the applicability domain of the model	To assess the applicability domain (AD) of the models we proposed a comprehensive approach where different AD assessment methods are combined to enhance the confidence of stakeholders in the produced predictions.<br>• In the bounding box (or range-based) method the interpolation space is considered the hyper-box defined by the range of minimum and maximum selected descriptor values.<br>• In the leverage method, the leverage values h -which are the diagonal elements of the Hat matrix- reflect the similarity of the validation or untested samples to the training set (distance from the training set's centroid) based on the descriptor values used in the model development. The limits of the AD are determined by the threshold leverage value h*. The prediction for a validation or untested NP is considered reliable if $h<h^*$.<br>• The last approach is based on the similarity of the closest training NPs to the query NP. In detail, this approach starts by applying the *k* nearest neighbour (*k*NN) methodology to the query NPs to assess the NP's local region in the hyperspace. For each query NP the *k* closest training NPs are selected based on the Euclidean distances between them, calculated considering the selected descriptors. Next, the cosine similarity between the query NP and each of the *k* training NPs is calculated and it is compared to a predefined threshold (sim *k*). If the similarity value of at least one of the *k* training NPs is below the threshold, the query NP is out of the AD limits and the prediction for this NP is considered unreliable.<br>Note that as the models are built using data for Ag, TiO_2 and CuO NPs, if they are used to predict the behaviour of other types of NPs (extrapolation), the differences between them and the training NPs should be considered.
5.2.	Method used to assess the applicability domain	Three different AD methodologies were applied namely the bounding box, the leverage, and the local similarity methods. The results of the three methodologies were summarised into a single weighted score value that classifies the overall predictions as "good", "moderate", and "poor".
5.3.	Software name and version for applicability domain assessment	KNIME Analytics Platform v.5.1.2, Enalos+ nodes.

5.4.	Limits of applicability	• Bounding box: An untested NP is outside the AD, if at least one of its descriptor values is out of the range of the limits of the corresponding descriptor defined by the training NPs.<br>• Leverage: h* = 0.440.<br>• Local similarity: *k* = 5 and sim*k* = 0.8.<br><br>For any query NP it is possible to assess its reliability based on the AD using a scoring system:<br>$score_i = w_{bb} \cdot AD_{bb,i} + w_{lev} \cdot AD_{lev,i} + w_{sim} \cdot AD_{sim,i}$<br>Where, $score_i$, is the combined reliability score of the ith query NP, w_{bb}, w_{lev}, and w_{sim} are the weighting factors of the bounding box, leverage and similarity AD methods respectively, and $AD_{bb,i}$, $AD_{lev,i}$, and $AD_{sim,i}$ are binary variables indicating whether the ith query NP is inside (value of 1) or outside (value of 0) the AD limits of the model according to the three AD methods.<br>The proposed values for each weighting factor are: $w_{bb} = 0.2$, $w_{lev} = 0.3$, and $w_{sim} = 0.5$.<br>Finally, the overall reliability of the prediction is proposed to be defined as follows:<br>$score_i < 0.5 \rightarrow Poor\ reliability$<br>$score_i = 0.5 \rightarrow Moderate\ reliability$<br>$score_i > 0.5 \rightarrow Good\ reliability$<br><br>Note that as the models are built using data for Ag, TiO_2 and CuO NPs, if they are used to predict the behaviour of other types of NPs (extrapolation), the differences between them and the training NPs should be considered.
**6**	**Defining goodness-of-fit and robustness (internal validation) – OECD Principle 4: "APPROPRIATE MEASURES OF GOODNESS-OF-FIT, ROBUSTENESS AND PREDICTIVITY"**	**PRINCIPLE 4: "APPROPRIATE MEASURES OF GOODNESS-OF-FIT, ROBUSTENESS AND PREDICTIVITY". PRINCIPLE 4 expresses the need to perform validation to establish the performance of the model. GOODNESS-OF-FIT and ROBUSTNESS refer to the internal model performance.**
6.1.	Availability of the training set	The training set is available as a supporting information file of the publication: Varsou *et al.* In Silico Assessment of Nanoparticle Toxicity Powered by the Enalos Cloud Platform: Integrating Automated Machine Learning and Synthetic Data for Enhanced Nanosafety Evaluation, *Computational and Structural Biotechnology Journal*, 2024. and at the NanoPharos DB (https://db.nanopharos.eu/Queries/Datasets.zul?datasetID=16).
6.2.	Available information for the training set	Nanomaterials dataset including physicochemical characterisation of the NPs and data on their *in vitro* toxicity to HepRG cells.
6.3.	Data for each descriptor variable for the training set	The training set is available as a supporting information file of the publication: Varsou *et al.* In Silico Assessment of Nanoparticle Toxicity Powered by the Enalos Cloud Platform: Integrating Automated Machine Learning and Synthetic Data for Enhanced Nanosafety Evaluation, *Computational and Structural Biotechnology Journal*, 2024. and at the NanoPharos DB (https://db.nanopharos.eu/Queries/Datasets.zul?datasetID=16).
6.4.	Data for the dependent variable for the training set	The training set is available as a supporting information file of the publication: Varsou *et al.* In Silico Assessment of Nanoparticle Toxicity Powered by the Enalos Cloud Platform: Integrating Automated Machine Learning and Synthetic Data for Enhanced Nanosafety Evaluation, *Computational and Structural Biotechnology Journal*, 2024. and at the NanoPharos DB

		(https://db.nanopharos.eu/Queries/Datasets.zul?datasetID=16).
6.5.	Other information about the training set	The training set comprises of 57 NP treatments randomly selected from the pool of the original NP treatments using stratified sampling. The training NP treatments are categorised as follows: 38 "Low effect" and 19 "High effect".
6.6.	Pre-processing of data before modelling	Considering the class imbalance (66% "Low effect" vs. 34% "High effect" treatments) in the training set, the minority class ("High effect") was oversampled to ensure that the number of treatments for each endpoint class is approximately equal, by employing the ADASYN methodology using *k*=5 neighbours. After oversampling the training set reached the 75 NP treatments (also available with the rest of the dataset). The training data were fed to a low variance filter (low variance threshold equal to 0.2) and to a Spearman's rank correlation coefficient filter (correlation threshold equal to 0.95), to remove non-essential descriptors. Variable selection was performed next according to §4.4.
6.7.	Statistics for goodness-of-fit	Statistics on the internal test set.<br>**Metric** – **Value**<br>Accuracy – 0.95<br>MCC – 0.90<br>Recall (sensitivity) – 1.00<br>Specificity – 0.92<br>Precision – 0.88<br>Cohen's Kappa – 0.89
6.8.	Robustness - Statistics obtained by leave-one-out cross-validation	Robustness – Statistics obtained by leave-one-out cross-validation (training set):<br>**Metric** – **Value**<br>Accuracy – 0.88<br>MCC – 0.76<br>Recall (sensitivity) – 0.92<br>Specificity – 0.84<br>Precision – 0.85<br>Cohen's Kappa – 0.76
6.9.	Robustness - Statistics obtained by leave-many-out cross-validation	Robustness – Statistics obtained by five-fold cross-validation (random selection, training set):<br>**Metric** – **Value**<br>Accuracy – 0.85<br>MCC – 0.73<br>Recall (sensitivity) – 0.97<br>Specificity – 0.74<br>Precision – 0.78<br>Cohen's Kappa – 0.71
6.10.	Robustness - Statistics obtained by Y-scrambling	Statistics (of the test set) for 10 iterations with scrambled endpoint values:<br>**Randomisation** – **Accuracy** – **MCC**<br>1 – 0.25 – -0.42<br>2 – 0.45 – -0.10<br>3 – 0.35 – -0.21

		4 0.65 0.31<br>5 0.60 0.12<br>6 0.50 0.10<br>7 0.60 0.32<br>8 0.40 -0.24<br>9 0.55 0.10<br>10 0.60 0.18
6.11.	Robustness - Statistics obtained by bootstrap	Not applicable
6.12.	Robustness - Statistics obtained by other methods	Not applicable
**7**	**Defining predictivity (external validation) – OECD Principle 4: "APPROPRIATE MEASURES OF GOODNESS-OF-FIT, ROBUSTENESS AND PREDICTIVITY"**	**PRINCIPLE 4: "APPROPRIATE MEASURES OF GOODNESS-OF-FIT, ROBUSTENESS AND PREDICTIVITY". PRINCIPLE 4 expresses the need to perform validation to establish the performance of the model. PREDICTIVITY refers to the external model validation. Section 7 can be repeated (e.g., 7.a, 7.b, 7.c, etc) as many times as necessary if more validation studies need to be reported in the QMRF.**
7.1.	Availability of the external validation set	The blind set is available as supporting information file of the publication: Varsou *et al.* In Silico Assessment of Nanoparticle Toxicity Powered by the Enalos Cloud Platform: Integrating Automated Machine Learning and Synthetic Data for Enhanced Nanosafety Evaluation, *Computational and Structural Biotechnology Journal*, 2024. and at the NanoPharos DB (https://db.nanopharos.eu/Queries/Datasets.zul?datasetID=16).
7.2.	Available information for the external validation set	Nanomaterials dataset
7.3.	Data for each descriptor variable for the external validation set	The blind set is available as supporting information file of the publication: Varsou *et al.* In Silico Assessment of Nanoparticle Toxicity Powered by the Enalos Cloud Platform: Integrating Automated Machine Learning and Synthetic Data for Enhanced Nanosafety Evaluation, *Computational and Structural Biotechnology Journal*, 2024. and at the NanoPharos DB (https://db.nanopharos.eu/Queries/Datasets.zul?datasetID=16).
7.4.	Data for the dependent variable for the external validation set	The blind set is available as supporting information file of the publication: Varsou *et al.* In Silico Assessment of Nanoparticle Toxicity Powered by the Enalos Cloud Platform: Integrating Automated Machine Learning and Synthetic Data for Enhanced Nanosafety Evaluation, *Computational and Structural Biotechnology Journal*, 2024. and at the NanoPharos DB (https://db.nanopharos.eu/Queries/Datasets.zul?datasetID=16).
7.5.	Other information about the external validation set	Blind set with 33 NP treatments appended.
7.6.	Experimental design of test set	Random-stratified selection of NP treatments before modelling (30% of the original set).
7.7.	Predictivity - Statistics obtained by external validation	**Metric** **Value**<br>Accuracy 0.88<br>MCC 0.75<br>Recall (sensitivity) 0.91<br>Specificity 0.86<br>Precision 0.77<br>Cohen's Kappa 0.74

7.8.	Predictivity - Assessment of the external validation set	The blind set is sufficiently large and as a stratified sampling technique was applied for its selection, it is ensured that the class distribution ("Low effect"/ "High effect") in the sets is representative of the original data. It was also assessed whether the blind set treatments were inside the AD limits and the results can be found in the supporting information file of the publication: Varsou *et al.* In Silico Assessment of Nanoparticle Toxicity Powered by the Enalos Cloud Platform: Integrating Automated Machine Learning and Synthetic Data for Enhanced Nanosafety Evaluation, *Computational and Structural Biotechnology Journal*, 2024.
7.9.	Comments on the external validation of the model	It is noted that considering that the modelling was performed inside an automated-ML scheme, the final selection of the model was based on the performance on an internal test set (statistics presented in §6.7). The blind set was used as an external validation set. For more information, please refer to the relevant publication: Varsou *et al.* In Silico Assessment of Nanoparticle Toxicity Powered by the Enalos Cloud Platform: Integrating Automated Machine Learning and Synthetic Data for Enhanced Nanosafety Evaluation, *Computational and Structural Biotechnology Journal*, 2024.
**8**	**Providing a mechanistic interpretation - OECD Principle 5: "A MECHANISTIC INTERPRETATION, IF POSSIBLE"**	**PRINCIPLE 5: "A MECHANISTIC INTERPRETATION, IF POSSIBLE". According to PRINCIPLE 5, a (Q)SAR should be associated with a mechanistic interpretation, if possible.**
8.1.	Mechanistic basis of the model	The selection of the concentration is rather obvious, considering that the data used for modelling are derived from dose-response experiments and thus, concentration values outline the experimental conditions. The selected atomistic descriptors include the difference of the average potential energy between the core and the shell atoms. The average potential energy group of descriptors expresses the stability of the particles (e.g., lower average potential energy values of the atoms correspond to more stable structures), while the common neighbour parameter (CNP) is a useful indicator of the local crystal structure around an atom that can be used to determine whether the atom is located in a perfect lattice, at a surface, or is part of a local defect. The average coordination number expresses the number of neighbouring atoms for a single atom. Every atom that is less than a user-defined distance away from an adjacent atom is considered a neighbouring atom. Descriptors defined as differences of the descriptors of the core and surface atoms are very informative of the NPs surface reactivity: if there is a significant difference in the average number of neighbouring atoms between core and shell (surface) atoms, the surface of the NP is expected to be highly reactive as there will be a significant number of unterminated bonds which could be recreated through reaction of the surface atoms with external atoms for example, of surrounding biomolecules. Therefore, higher values of these descriptors indicate highly reactive NPs. Finally, the log10 of all atoms of the NP is connected to the NP's size and lower values of this descriptor indicate smaller NPs which in general are more toxic than larger particles at an equivalent mass due to higher particle numbers and more internalisation. Nonetheless, it should be noted that these descriptors are calculated for uncoated NPs in vacuum and thus, a direct relationship between the NPs descriptors-reactivity and their toxicity cannot be established at this point. Computational study of the NP coatings, which is currently being developed, will further contribute to the understanding of the behaviour of NPs in biological media and thus, will enlighten the mechanisms that drive nanotoxicity.
8.2.	A priori or a posteriori mechanistic interpretation	A posteriori mechanistic interpretation.

8.3.	Other information about the mechanistic interpretation	Not applicable
**9**	**Miscellaneous information**	
9.1.	Comments	Not applicable
9.2.	Bibliography	Information on data generation: Joossens, E., Macko, P., Palosaari, T. *et al.* A high throughput imaging database of toxicological effects of nanomaterials tested on HepaRG cells. Sci Data 6, 46 (2019). https://doi.org/10.1038/s41597-019-0053-2<br>Information on Random Forests: Tin Kam Ho, "Random decision forests," *Proceedings of 3rd International Conference on Document Analysis and Recognition*, Montreal, QC, Canada, 1995, pp. 278-282 vol.1, doi: 10.1109/ICDAR.1995.598994.
9.3	Supporting information	Not applicable
